# Inferior Vena Cava Thrombosis in a Young Patient With COVID-19 Infection

**DOI:** 10.7759/cureus.24145

**Published:** 2022-04-14

**Authors:** Ali Rahman, Sura Alqaisi, Chad W Downing, Daniele J Kenny, William LiPera

**Affiliations:** 1 Internal Medicine, Northwell Health at Mather Hospital, Port Jefferson, USA; 2 Radiology, Mather Hospital, Port Jefferson, USA; 3 Family Medicine, Mather Hospital, Port Jefferson, USA; 4 Hematology/Oncology, Stony Brook University Hospital, Port Jefferson, USA

**Keywords:** corona virus disease, ivc thrombectomy, venous thromboembolism (vte), ivc filter, inferior vena cava thrombosis

## Abstract

Inferior vena cava thrombosis (IVCT) is a potentially fatal condition that may rarely occur in young patients with COVID-19 infection. This report describes a young adult female with a recent COVID 19 infection who presented with fever, bilateral flank pain, elevated inflammatory markers, and evidence of thrombosis in the inferior vena cava (IVC) on computed tomography (CT). The patient required treatment with anticoagulation therapy and catheter-directed thrombolysis, IVC filter placement, and mechanical suction-assist thrombectomy.

## Introduction

Inferior vena cava thrombosis (IVCT) is a rare condition that may develop in patients who are in a hypercoagulable state. COVID-19 infection has been known to induce hypercoagulability and, thus, may rarely result in the development of IVCT [1]. Only a few case reports of IVCT in young patients with COVID-19 infection can be found in the literature [1,2]. In this report, we present the case of a young patient who presented with a fever, bilateral flank pain, and elevated inflammatory markers. A diagnosis of IVCT was established based on an abdominopelvic computed tomography (CT) scan demonstrating a thrombus extending from the IVC to the common iliac veins, right external iliac vein, and right renal vein. The thrombus extended into the left femoropopliteal vein later in the disease course, despite the administration of anticoagulation therapy.

## Case presentation

A 23-year-old woman with a past medical history of migraines and a recently diagnosed COVID-19 infection presented to the emergency department of Northwell Health hospital, NY, with right flank pain that started two to three days prior. The patient reported that she had developed a cough and sore throat almost two weeks ago and had tested positive for COVID-19 infection six days ago. Her respiratory symptoms have subsequently subsided. On presentation, the patient was tachycardic, and her complete blood count was significant for mild leukocytosis. Urine analysis was positive for pyuria, bacteriuria, hematuria, and proteinuria. Blood and urine cultures were negative. The patient was treated for pyelonephritis and was discharged. 

**Figure 1:**
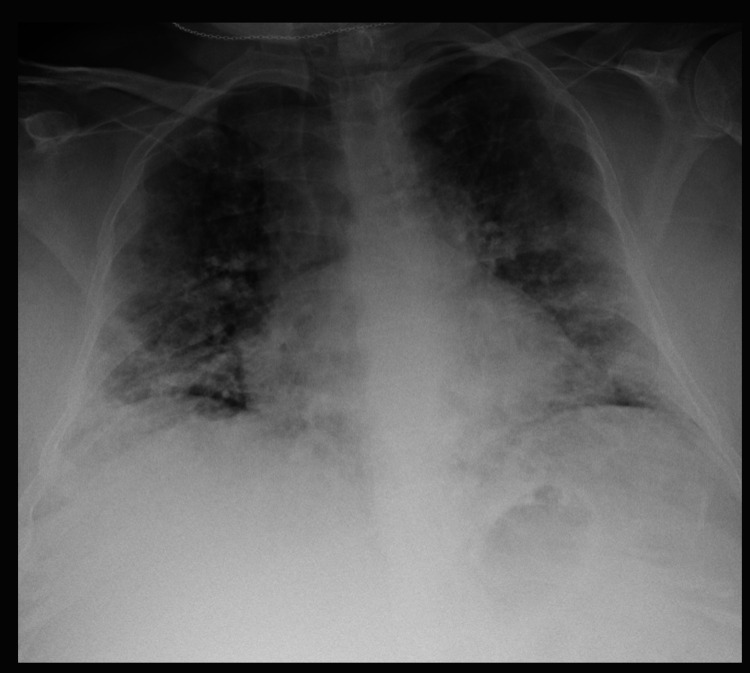
CXR demonstrates diffuse bilateral airspace opacities, new when compared to prior from approximately five months ago.

**Figure 2:**
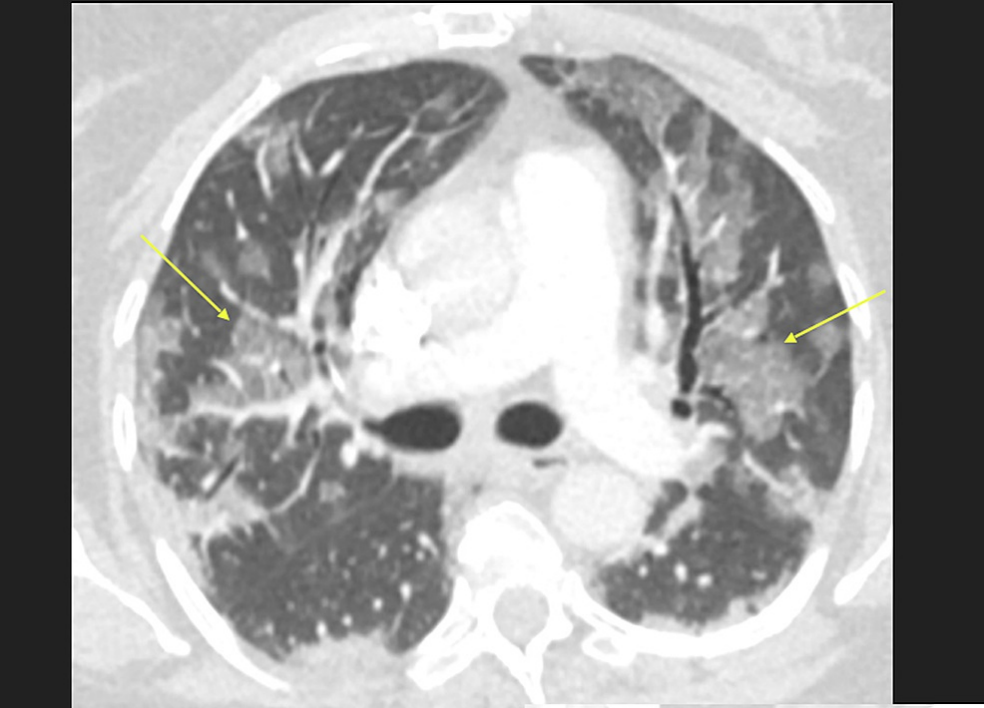
Axial CT image shows bilateral ground glass opacities (arrows) with involvement of all five lung lobes.

**Figure 3:**
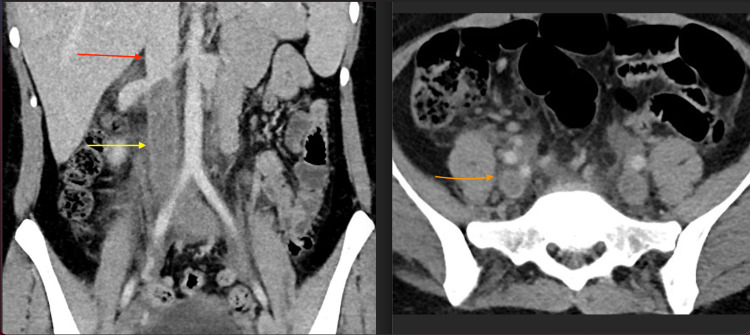
Coronal (left) and axial (right) contrast enhanced CT images show extensive thrombus within the infrarenal IVC (yellow arrow) extending into the right and left common iliac veins (orange arrow). The suprarenal IVC is patent (red arrow).

The patient returned to the emergency department three days later with a fever and bilateral flank pain. Her temperature was 101 °F, her blood pressure was 125/77 mmHg, her respiratory rate was 28 cycles/minute, and her heart rate was 128 beats/min. Her physical exam was unremarkable except for bilateral coarse crackles at her lung bases. A complete blood count revealed leukocytosis with lymphopenia. She was positive for COVID-19 and had an elevated erythrocyte sedimentation rate, serum ferritin, D-dimer, and fibrinogen levels. Lab results are in Table 1. Troponin T levels were unremarkable, and a normal sinus rhythm pattern was seen on electrocardiography. A chest X-ray showed bilateral airspace opacities (Figure 1). The CT chest showed bilateral ground-glass opacities (Figure 2). Echocardiography showed an ejection fraction of 69% with normal left ventricular (LV) diastolic function and right ventricular (RV) systolic pressure. An abdominopelvic computed tomography (CT) scan showed a thrombus extending from the inferior vena cava (IVC) to the common iliac veins, right external iliac vein, and right renal vein (Figure 3). A duplex scan was negative for lower extremity deep venous thrombosis (DVT).

**Table 1:** Laboratory results

Labs	Values	Normal range	Units
WBC	13.5	4.5–11	*10^9^/L
Hemoglobin	14.4	12–16	g/dl
Platelets	160	130–400	*10^9^/L
Neutrophils	85	40–60	%
Lymphocytes	8.2	20–40	%
Monocytes	5	1.7–9.3	%
Eosinophils	1	0–5	%
Basophils	0.8	0–3	%
Sodium	137	137–145	mmol/L
Potassium	4	3.5–5.2	mmol/L
Chloride	99	98–107	mmol/L
Carbone dioxide	22	22–30	mmol/L
BUN	11	7–17	mg/dl
Creatinine	0.9	0.52–1.04	mg/dl
ESR	213	0–29	mm/hr
D-dimer	3833	<250	ng/ml
Ferritin	74	12–50	ng/ml
Fibrinogen	>700	200–400	mg/dl
SAR-COV 19 PCR	Positive	Negative	

**Figure 4:**
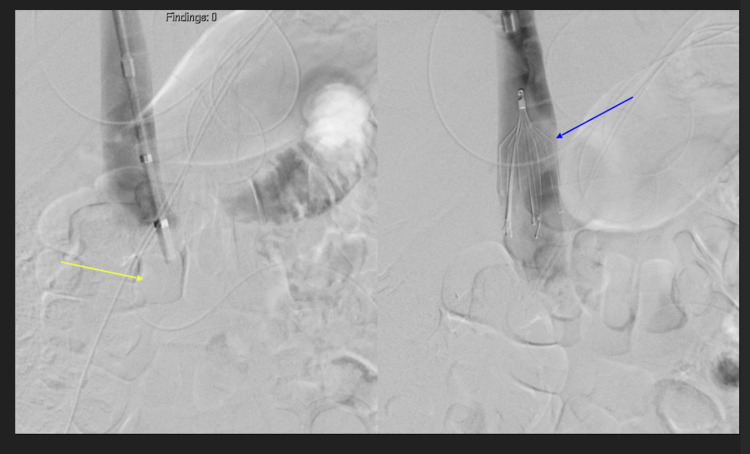
Initial venogram (left) shows complete occlusion of the infrarenal IVC (yellow arrow). Placement of IVC filter (blue arrow, the procedure was ended early after partial thrombectomy (not shown) due to blood loss.

The patient was administered enoxaparin 80 mg subcutaneous, ketorolac 30 mg IV, and ondansetron 4 mg IV in the emergency department and was admitted to the hospital for the management of the extensive thrombosis in the vessels mentioned above secondary to COVID-19. Her outpatient medicines included oral contraceptive pills, which were stopped. Heparin drip at a rate of 1400 units/hour was initiated. Moreover, given her recent positive urinalysis, she received ertapenem 1 g once a day for three days. A hypercoagulability workup was performed and was negative for heterozygous factor V Leiden mutation, negative antiphospholipid syndrome (anticardiolipin IgM was slightly elevated (13U/ml)), and increased factor VII activity (251%). The patient underwent transjugular inferior vena cava (IVC) filter placement (Bard Denali filter/suprarenal location; Figure 4). Her activated partial thromboplastin time (aPTT) remained in the sub-therapeutic range (40-50 sec).

**Figure 5:**
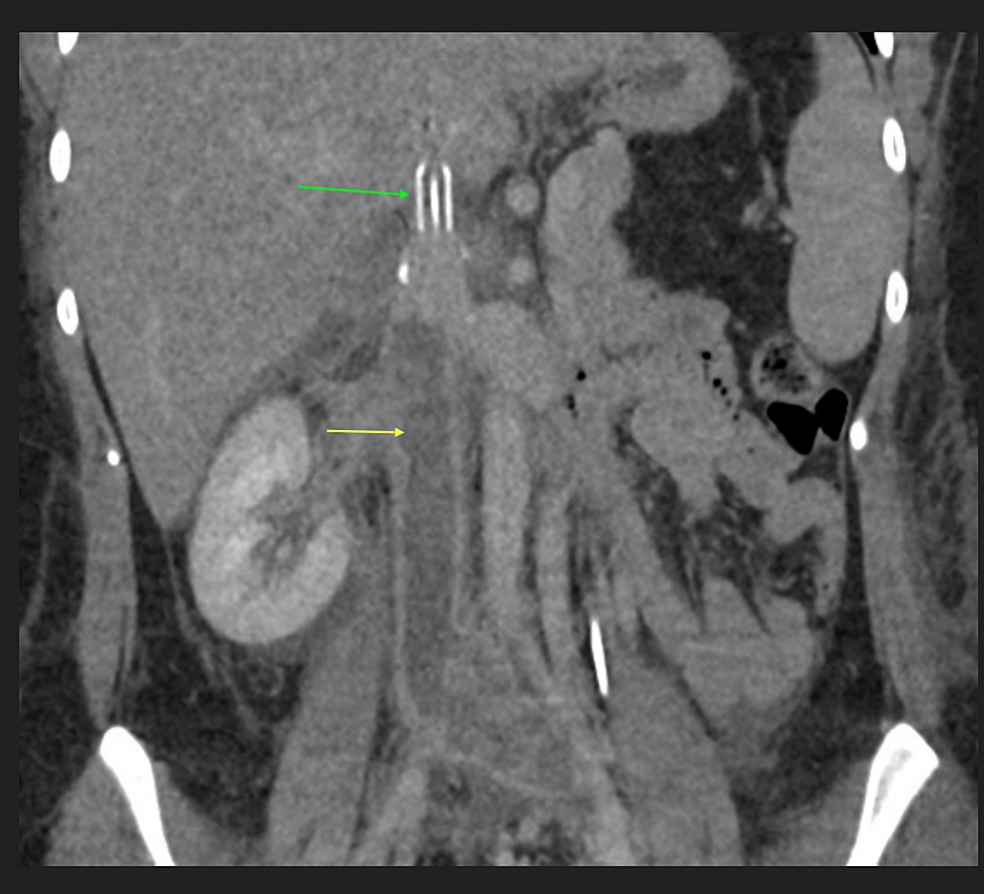
Contrast-enhanced coronal CT image of the abdomen shows re-thrombosis of the IVC after partial thrombectomy and placement of IVC filter (partially visualized, green arrow), the yellow arrow shows the infra-renal IVC.

Two days later, the patient complained of left leg pain and soreness. A newly developed DVT was found in the left femoropopliteal vein (Figure 5). At this point, the heparin drip was switched to an argatroban drip. The patient also underwent multiple mechanical suction-assist thrombectomies of the IVC, bilateral common iliac veins, right renal vein, and left femoropopliteal vein, and sufficient patency was established. She also received catheter-directed alteplase for three days and was switched from argatroban drip to apixaban 10 mg oral every 12 hours. Her discharge plan for anticoagulation consisted of apixaban 10 mg oral every 12 hours for 7 days and then 5 mg every 12 hours until a hematological assessment was performed on an outpatient basis.

## Discussion

Severe acute respiratory distress syndrome coronavirus 2 (SARS-CoV-2) is a novel coronavirus that is responsible for causing coronavirus disease 2019 (COVID-19). Although respiratory compromise is the primary feature of this condition, it has been found that COVID-19 may also result in the development of hypercoagulability and thromboembolism. According to a Dutch study, a high incidence of thrombotic complications was observed in critically ill patients with COVID-19 admitted to the intensive care unit (ICU). In this study, venous thromboembolism (VTE), including DVT and pulmonary embolism (PE), was confirmed in 27% (95% CI, 17-37%) of the patients, while arterial thrombotic events were confirmed in 3.7% (95% CI, 0-8.2%) of the patients [1]. In another study involving 184 critically ill ICU patients with COVID-19, the incidence of thrombotic complications was found to be 31% [2]. In contrast, the incidence of thrombotic complications in non-hospitalized patients who have COVID-19 with mild or no symptoms appears to be very low. Nevertheless, several such cases have been reported in the literature [3]. For instance, Uppuluri and Shapiro have reported the case of a non-hospitalized patient with COVID-19 who did not receive VTE prophylaxis and developed symptoms of PE [4]. Similarly, in a retrospective cohort study conducted by Trimaille et al., the incidence of VTE in non-critically ill patients with COVID-19 infection was determined to be around 17% [5].

The exact pathophysiology of COVID-19-induced hypercoagulability is not completely understood. However, all three components of Virchow’s triad, i.e., venous stasis, endothelial injury, and prothrombotic changes, appear to be involved. Endothelial injury is evident from the virus's direct invasion of endothelial cells via the angiotensin-converting enzyme 2 (ACE-2) receptors and increased angiogenesis. The release of inflammatory cytokines (e.g., interleukin (IL)-6) and various acute phase reactants and the activation of the alternate and lectin complement pathways may also result in endothelial injury. Stasis may occur due to immobilization in hospitalized patients, while increased coagulation may be seen due to elevated prothrombotic factors, such as fibrinogen, D-dimers, factor VIII, and von Willebrand factor (vWF) [6]. It has also been proposed that COVID-19 infection may establish an inflammatory and prothrombotic microenvironment leading to more severe thrombotic events in asymptomatic factor V Leiden heterozygotes, as was likely the case with our patient [7].

Patients with COVID-19 have been found to develop thrombotic complications in various locations, including the venous, arterial, and microvascular systems. IVCT has also been reported in a few patients with COVID-19 infection. For example, in a recently published study that evaluated 145 previously healthy, non-critically ill, young adults with COVID-19, 38 thrombotic events were identified, which included three cases of IVCT [7].

IVCT is a rare clinical entity that is considered to be a subset of DVT. The condition is often under-recognized as it is often not pursued as a primary diagnosis. According to the United States National Hospital Discharge Survey, from the years 1979 to 2005, the incidence of vena cava thrombosis (presumed to be predominantly IVCT) was found to be only 1.3% among all hospitalized patients diagnosed with venous thrombosis [8]. The clinical presentation of patients with IVCT may vary depending on the extent and site of thrombus formation within the IVC. Typical symptoms include pain, swelling, and cramping of the lower limbs combined with dilation of superficial abdominal veins. Multiple imaging modalities, such as IVC duplex ultrasound, computed tomography (CT), magnetic resonance imaging (MRI), and catheter venography, can be used to visualize a potential thrombus in the IVC. Due to its low-risk profile, duplex ultrasound is usually the first test to be performed. The location of the clot may also be detected using the CT scan. However, MRI is the most reliable non-invasive tool that can determine the presence and extent of the thrombus. Direct catheter venography may be used to establish a definitive diagnosis [9,10].

The initial management of patients with IVCT consists of anticoagulation with heparin, which can then be switched to warfarin or a newer generation anticoagulant. Catheter-directed thrombolysis or thrombectomy may be used if the IVCT is acute (<14 days) or subacute (15-28 days), and if the patient is not at a high risk of bleeding. Endovascular interventions, such as percutaneous transluminal angioplasty with stenting, may also be of benefit, particularly if the IVCT is chronic (>28 days) [9,10,11]. Our patient was started on heparin in the emergency department. However, multiple studies have shown that despite the standard anticoagulation prophylaxis, there remains a high incidence of thrombotic complications in patients with COVID-19 infection [1,12,13]. This was also true for our patient, who, while on therapeutic anticoagulation for almost a week, not only had persistent thrombosis in the inferior vena cava, common iliac veins, and right renal vein but also demonstrated extension of the thrombus into the left femoropopliteal vein. She consistently had sub-therapeutic levels of aPTT (<50 seconds) while on heparin, and hence, it was switched to argatroban, and aPTT levels in the desired range (55-88 seconds) were achieved. In addition, we also attempted catheter-directed thrombolysis with alteplase, which is a tissue plasminogen activator (tPA).

Inferior vena cava (IVC) filters are small mechanical devices that are placed in the IVC to stop the thrombus from migrating into the cardiopulmonary system, thus preventing the development of PE. However, the prophylactic use of a temporary IVC filter before performing mechanical thrombectomies in patients with DVT remains controversial due to a lack of prospective studies [14]. Nevertheless, IVC filter implantation in patients with COVID-19 infection and concurrent DVT has been reported to successfully prevent the development of fatal PE in several cases [15-17]. Our patient also underwent IVC filter placement and later had multiple thrombectomies, and enough patency was established in her affected veins.

It has been observed that increased levels of inflammatory markers, such as white blood cell (WBC) count, C-reactive protein (CRP), erythrocyte sedimentation rate (ESR), D-dimers, fibrinogen, IL-6, and IL-10, are associated with increased severity of COVID-19 disease [18-20]. Hence, these markers can be used for the early identification as well as the prediction of disease progression.

## Conclusions

Inferior vena cava thrombosis is a subset of DVT that has been reported infrequently in the literature. COVID-19 is a pro-inflammatory state that may increase the risk of VTE, including IVCT, in young, non-hospitalized adults. If left untreated, the condition can result in serious complications such as post-thrombotic syndrome (90%), venous claudication (45%), pulmonary embolism (30%), and venous ulceration (15%). In addition, as compared to the mortality rate of deep vein thrombosis confined to the lower extremities, the mortality rate of IVCT has been reported to be two-fold higher. Therefore, early recognition of the condition and initiation of anticoagulation therapy while evaluating the patient in the emergency department is critical. Elevated inflammatory markers, such as WBC count, ESR, CRP, D-dimers, and fibrinogen, may be used to establish an early diagnosis. Furthermore, physicians should be aware of the fact that thrombotic complications may occur in young patients several days after acquiring the COVID-19 infection. Finally, IVCT may persist despite the use of anticoagulation therapy, and additional interventions, such as catheter-directed thrombolysis, IVC filter implantation, and IVC thrombectomy may be required in severe cases.
